# Hospital and Institutional News

**Published:** 1917-08-18

**Authors:** 


### HOSPITAL AND INSTITUTIONAL NEWS.

### SIR WILLIAM WATSON CHEYNE, M.P.

We congratulate Sir William Watson Cheyne on his election as the Member of Parliament for the Universities of Edinburgh and Saint Andrews. Sir William is widely known as one of the foremost surgeons of the present day; he studied under Lister in Edinburgh, and when Lister came to London to take up the post of surgeon to King's College Hospital, Mr. Watson Cheyne accompanied him, and only a comparatively short time ago he resigned the post of senior surgeon at that hospital. He has filled the post of President of the Royal College of Surgeons of England for two years, and resigned the post in July of this year. Sir William should prove a very valuable addition to the House of Commons, where there are not so many doctors as there should be, for medical matters are always arising and the House stands in great need of competent advice. It is curious that there are not more members of the medical profession in the House of Commons in this country, for in many foreign Legislatures the doctors as a rule form no small proportion of the whole.

### THE GENERAL HOSPITAL, BIRMINGHAM, AND THE PLENUM SYSTEM.

Hygiene has made such strides in the last thirty years in the enforcement of its principles, and proved of such inestimable value in saving life and diminishing illness amongst the armies of the Allies in the field, that it is instructive to read a correspondence in the *Birmingham Daily Post* upon open windows at the General Hospital. It is remarkable that the plenum system, which system was the idiosyncrasy of the late Mr. William Henman, the architect, should still be suffered at the General Hospital, the great voluntary hospital of Birmingham. We say remarkable, because the late Mr. Henman, when he had his plans ready, brought them with him to a consultation which took place in London, by his wish, with the late Dr. John S. Billings, the great American authority on hospital construction, and Sir Henry Burdett, on September 20, 1894. The advice given to Mr. Henman at this consultation was embodied in the following Report on the Plans of the New General Hospital, Birmingham; it bears the above date, and was prepared immediately after the interview: "Mr. William Henman, the architect of the new Birmingham General Hospital, having very kindly submitted and explained his plans, including the new system of heating and ventilation, for our information, we beg to express the following opinion in regard to them. Having regard to the limited site, the plan appears to be, on the whole, good. Experience has proved that the placing of an out-patient department below a large general ward, the suggested arrangement of the consulting and retiring rooms, together with the location of the wards for burns in the same block as those devoted especially to women and other special cases, are not the best arrangements that could be proposed. We understand, however, that Mr. Henman's instructions did not permit him to plan the out-patient department differently. The proposed plan of heating and ventilating the hospital by indirect radiation and electric fans, relying entirely upon these methods for securing pure air of a proper temperature throughout the whole building, is theoretically practicable, but will probably prove to be unnecessarily costly, since it requires the use of mechanical ventilation by electric motors day and night throughout the whole year. We shall watch the working out of the Birmingham experiment with great interest, but we fear experience will prove that when the cost of continuing the system at a high state of efficiency is realised, protests will be made against so serious an annual outlay, which will lead to attempts to reduce the cost, and so to gradually lessen the efficiency of the ventilation and heating arrangements, until they will become ineffective and unsatisfactory. It will then be difficult to change to the much cheaper system in use in the best modern hospitals, in which the required circulation of air is effected during a considerable part of the year by open windows and heated vertical shafts, because in the plan proposed all windows are hermetically sealed, and shafts of sufficient area are not provided. We have not been asked for an opinion or suggestions, and merely make this as a memorandum of our opinion, while the matter is yet fresh in our minds.—London, September 20, 1894. (Signed) John S. Billings, Henry C. Burdett."

### THE EXISTING EVILS AND THEIR REMEDY.

This report was not published in the absence of a request from the hospital authorities for such an opinion, because the signatories did not want to be in the position of gratuitously attacking the architect. They felt that after the hospital had been running they would see how far their judgment proved correct, and, should a special demand at any time arise, it might then usefully be published. The correspondence now proceeding in the *Birmingham Daily Post*, and especially the letter of "M.D.," we take as evidence that such a demand has now arisen, and in the interests of the patients and the staff of the Birmingham General Hospital Sir Henry Burdett has requested us to publish this report. The correspondence in the *Daily Post* was commenced by an architect whose daughters are nurses, on the ground that the substitution of open windows would "lead to a cheapening of the cost of maintenance, through the quicker recovery of the patients and the better health of the permanent staff." "M.D." writes:—"This complaint is entirely justified. Doctors, nurses, patients, and everybody concerned, complain of the evil effects of the system, which, however perfect in theory, is absolutely bad in practice." He confirms the forecast of the experts as given in the report by declaring, at the end of twenty years'

experience, that:—"The value of fresh air is proved beyond question, not only in hospital practice, but by every soldier, whose ruddy face and healthy appearance amaze everybody. It seems strange that the one place from which fresh air is rigidly excluded is the magnificent building devoted to the restoration of health. The truth is that this system at the General Hospital has long been acknowledged as a mistake, and would have been cast aside but for the expense involved. Here, then, is a magnificent opportunity for some rich benefactor." Rich benefactor or not, we are confident that the citizens of Birmingham as a whole, in view of the facts as they stand, will at once provide the relatively few thousand pounds required to free both patients and staff, who have suffered long enough from the evil effects of a system which, but for the idiosyncrasy of one man, they might never have been called upon to endure. The children have suffered more severely than adults by the application of the plenum system to hospital wards, where in one hospital, years ago, we found the children almost asphyxiated for want of necessary oxygen, and took steps which mercifully ended the plenum system there, and the children's miseries. "H. C. H." gives one startling reason for these evils:—"It has been overlooked that the small quantity of air is naturally dissolved in the water when passing through water screens in the proportion of oxygen 100 to nitrogen 187, but the original air consisted of oxygen 100 to nitrogen 400, which means an excessive quantity of nitrogen passing into the building." (Poor children! And what of the staff and patients?) "This is one of the imperfections of the plenum system." The Birmingham General Hospital should be freed from this and other evils attending its use, and revert to fresh-air ventilation and open windows without a moment's further avoidable delay. We understood that years ago the plenum system was abolished in the case of the quarters of the residents and the nursing staff. Is this not so?

### BONE-SETTERS AND THE WOUNDED.

For some little time there have been attempts to utilise the services of Mr. Barker, the bone-setter, for the treatment of the wounded, and a number of members of the House of Commons have united for the purpose of obtaining official recognition of his treatment. The matter has been again brought up in the House, when Mr. Peto, the member for Devizes, moved to reduce the vote by £100 in order to call attention to the refusal of the War Office to allow wounded soldiers to be treated by "manipulative surgery." Sir William Watson Cheyne made his maiden speech in the debate, and he pointed out that if they were to permit the utilisation of the services of one unqualified practitioner, they would not be able to refuse to admit anyone claiming to be able to treat disease and injury. He mentioned that a tuberculous joint might be healed by careful attention and rest, but if it were treated with violence the result would be disastrous; and so with many other conditions. It must not be forgotten that there is nothing peculiar in "manipulative surgery"; the methods employed by bone-setters are those which are known to all surgeons, though some may appreciate their importance more than others. We hear, indeed, of the successes of bone-setters, but we do not hear of their failures, which are many, and several surgeons have seen grievous examples of the harm that has resulted from attempting forcibly to bend diseased joints. Stiffness due to adhesions in a joint after injury or disease can often be almost instantaneously remedied by forcible flexion, and these form the class of cases of which we hear so much. There is no secret in the matter and equally good results are being obtained daily by surgeons, yet nothing is said of them. Any officer or private may apply for treatment to a bone-setter, but he does it on his own responsibility. There is no insuperable difficulty in a bone-setter obtaining a medical qualification if he is willing to undergo the requisite training, and therefore there can be no excuse for his remaining unqualified.

### MEDICAL HONOURS AND PROMOTIONS.

Recent issues of the *London Gazette* contain particulars of numerous honours conferred on members of the medical profession. Five members of the R.A.M.C. have received the C.B. (Military Division). One, Temporary Surgeon-General M. T. Yarr, C.B., F.R.C.S.I., has been granted the K.C.M.G. The C.M.G. has been conferred on ten members of the R.A.M.C., and the same honour has also been conferred on three members of the New Zealand Medical Force. Thirty-two Lieutenant-Colonels R.A.M.C. have been promoted to be Brevet Colonels; twenty-nine Majors R.A.M.C. have been made Brevet Lieutenant-Colonels, and twenty-eight Captains R.A.M.C. have been promoted to the rank of Brevet Major.

### SOME OF THE RECIPIENTS.

Amongst the recipients is Temp. Lieut.-Col. W. L. W. Marshall, R.A.M.C., commanding the Huddersfield War Hospital, who is in charge of over 2,000 beds in that hospital and its auxiliaries. Col. Marshall, who is awarded the C.M.G., has been in command of war hospitals in the Huddersfield locality since they were established towards the close of 1915, and was gazetted lieutenant-colonel on September 1, 1915. Twice previously he has been "mentioned" for distinguished services in connection with the war. He bears a high reputation as a surgeon. Surgeon Lieut.-Col. D. Hepburn, V.D., who has also been awarded the C.M.G. following two "mentions," commands the 3rd Western General Hospital, Cardiff. Colonel Hepburn, who is a voluminous writer on medical subjects, came to Cardiff in 1903 to take up important work at the University College of Wales, where, when the war broke out, he was Professor of Anatomy and Dean of the Faculty of Medicine. The rank of Brevet Colonel has been conferred upon a number of R.A.M.C. officers, including Lieut.-Col. H. L. Battersby, who is in charge of the military hospitals in Nottingham; Major George W.

Watson, one of the physicians at Leeds Infirmary, who has done valuable work at Beckett's Park Military Hospital, Leeds; and Lieut.-Col. G. Sims Woodhead, Professor of Pathology at Cambridge University.

### A PROPOSED NEW AMBULANCE CORPS.

In consequence of a letter from the Rev. Percy Jackson to the *Spectator*, in which the writer suggested the formation of a parsons' ambulance corps, the Bishop of London repeats a proposal which he says he made unsuccessfully two years ago. Such a corps, he declares, "was then said to be impracticable, because those clergy who enlisted in the R.A.M.C. became ordinary privates under orders, and had no guarantee that they would not be employed as clerks or in other R.A.M.C. work far from the firing line—work which cannot be compared in importance with that they are now doing in their respective parishes." "If, however," the Bishop continues, "Mr. Jackson has information which leads him to suppose that a parsons' ambulance corps, or stretcher-bearing company, for part-time service is a practical possibility, I should be very glad to hear from him on the subject, as numbers of the younger clergy are only too anxious to share the dangers and privations which men of their own age in the Army are daily enduring." The formation of such special units is always a matter of difficulty, and we do not see why the clergy who enlist in the R.A.M.C. can expect preferential or different treatment from their fellows, since men of the new corps would have their necessary share of clerical and administrative work.

### THE PROTECTION OF HOSPITAL SHIPS.

The Germans and their allies have on many occasions destroyed by the torpedo hospital ships belonging to us, and as an excuse for this action they have alleged that we habitually send troops and munitions by hospital ships. Although this accusation is denied, the Germans merely repeat their tales, and as a result the precautions supposed to protect hospital ships, such as their complete illumination, have only served to send them more surely to their doom. An attempt is now being made to overcome the excuses of the Germans: a Spanish officer is to travel in every hospital ship, and he is to assure himself that no combatants and no munitions are on board. The Germans will, we are told, respect a hospital ship if it has this Spanish officer on board. We hope that the plan will prove successful, though we should not like to be too sure. An explanation which has been advanced to account for the present action of the Germans is that they are finding that their ruthless submarine policy is doing them harm by its effect on neutrals, and the effect it has in lessening the chance of peace for which they long ardently.

### THE LIMITS TO EDUCATIONAL CONVALESCENCE.

The lengths to which the training of cripples is going borders on the grotesque, and an intelligent observer, invited to admire an armless man in the act of shaving with his foot, cannot but feel that he is witnessing a necessity to be deplored rather than a feat to be applauded. Whether time will not show that the nervous strain involved in some of the more extraordinary of cripples' performances does not set a natural limit to their use is a question worth pondering on. But that time has not arrived, and what is called "educative convalescence" is being as rigidly enforced as what is known as "scientific management" in America. Indeed, the two, when pushed to extremes, have something in common, and their common something has a sinister air. Among the latest devices is that of setting cripples to teach each other. To-day we have learnt that the blind can lead the blind, and that only if they do not is the ditch an alarming probability. So long, however, as the "economic success" claimed by such training does not endanger a disabled soldier's pension, nor subject him to *laissez-faire* conditions of employment, the project is comparatively sane. Having registered a warning in regard to its possible abuses, let us consider briefly the process itself as carried on in an advanced manner in a Sussex colony.

### SOLDIERS AT SCHOOL.

At Chailey, in Sussex, the Heritage Craft Schools for Crippled Children began to receive two years ago batches of crippled soldiers. Each soldier, we learn, was furnished with two crippled boys as orderlies, and the infirmities of the orderlies were chosen to match those of the soldiers whom they attended. The children taught to the men the skill they had learnt. The organisation of workshops was soon extended. Artificial limbs were made on the premises, and what is asked for at present is for the extension of these workshops, and for a fitting room at one of the big hospitals to complete the scheme. Since such men can become self-supporting, it is important to insist that the wages so earned shall not in any way diminish the full pension to which the men are entitled. To exploit the cripples' skill would be a disgrace to the nation. As The Hospital pointed out long ago, "The disabled should stick to their pensions as fast as they formerly stuck to their guns, and the nation should be equally proud of them for doing it." At present there is accommodation for fifty soldiers at the Heritage Schools, but probably this number will be increased or the example followed in other parts of the country.

### AN OVER-SUBSCRIBED SPECIAL APPEAL.

Mr. J. H. Smethurst, the chairman of the Board of Warrington Infirmary, has often deserved well of his institution in the past because of the energy and resource which he has thrown into its affairs. His latest success has been to raise a special fund of £4,000 for the infirmary, which was over-subscribed in eight weeks, and we believe by this time has reached £5,000. The special departments which are being opened in connection with the out-patient unit are not only costly in themselves, but, in consequence of their success, make the extension of the accommodation for out-patients an urgent matter. Firms and individuals in the district have come forward generously. The

Guardians themselves gave a special donation, and their joint action shows how much appreciated is the work of an institution the authorities of which keep in close touch with the life of those they serve. Mr. Smethurst in his time has employed many methods of interesting the public in his institution. Some of his experiences have been described in The Hospital in the past. His reward is seen in the support which Warrington Infirmary never fails to receive in times of difficulty.

### THE SIZE OF CONVALESCENT HOMES.

The Derby and Derbyshire Convalescent Home at Matlock, which placed twenty-five out of its thirty-six beds at the disposal of the Northern (Military) Hospital, has had to rely on public generosity to make ends meet. The War Office allowance is 2s. per head per day, and the expense incurred has worked out at 3s. 1d. Having retained eleven beds for the use of civilians, sixty-four men and eighty-three women were received, together with 219 soldiers. There is a considerable amount of quiet, useful work going on among the smaller institutions of the country; and, so far as convalescent homes are concerned, there can be little doubt that the men have freer and more human surroundings in the smaller homes than in the more imposing buildings, where numbers and regulations crowd upon each other.

### THE LEICESTER HOME FOR SHELL-SHOCKED SOLDIERS.

The universal sympathy and the willingness to help the wounded soldier was evidenced at a recent meeting of the Town Council of Leicester, when the Mayor's application for the use of the Leicester Frith House and a portion of the estate as a home for discharged neurasthenic sailors and soldiers was considered. In moving the reception of the Estate Committee's report recommending that the application be granted, Alderman Sawday mentioned that his committee had offered the house, land, and garden at a rental of £150 per annum, and suggested that the Sanitary Committee and the Farms Committee be approached with a view to transferring the garden and such portion of the adjoining land now occupied by these committees as might hereafter be required for the purposes of the Home. It was not proposed at once to hand over the whole estate of 100 acres, but in the event of the need arising, he was sure the Council would be ready to do so. In the years to come, this exceedingly valuable estate might be used for a public park and the house as a museum or for other useful municipal purposes. The recommendation was readily accepted.

### MANCHESTER COUNCIL FOR VENEREAL DISEASES

The Lord Mayor of Manchester presided at a meeting on July 30 called for the purpose of forming a local branch of the National Council for Combating Venereal Disease. He explained that the meeting was held under the auspices of the City Council, and especially of the Sanitary Committee, with a view of disseminating information among the general public, so that the importance of these diseases and of their prevention might be more fully and widely recognised. The chief speaker was Sir Malcolm Morris, who was a member of the Royal Commission on Venereal Diseases. He laid stress on the economical side of the matter, for he pointed out that an enormous saving would be brought about by the prevention and early cure of these diseases, and that this saving would be not only in money but also in human life and suffering. He showed that an enormous amount of harm was done by venereal diseases to the general health of the community, and this was often due to the neglect of treatment in their early stages, and that trivial and quack remedies only served to hide the manifestations of the disease, so that the sufferers imagined that they were cured while only the symptoms and signs were hidden. The advance of our knowledge in the means of diagnosing venereal diseases, and even more in their treatment, had entirely changed the problems set to the medical profession, so that now these diseases were easily, rapidly, and thoroughly cured. He explained that Dr. Niven, the medical officer of health for Manchester, had drawn up a scheme, which was admirable in its details, and which provided for treatment free of cost, secret and humane. The scheme was entirely voluntary, and no compulsory notification was asked for, for it was thought that that would lead to many difficulties. Mrs. Gotto pointed out that an obstacle to the early treatment of venereal disease was the misconduct rule in many friendly societies, and she thought that Manchester was to be congratulated that at a recent conference it had led the way by passing a unanimous recommendation that this rule should be rescinded. A general committee was formed to arrange for the establishment of a branch of the National Council for Combating Venereal Disease.

### THIS WEEK'S DRUG MARKET.

Business continues quiet, with few remarkable features. Generally speaking, the upward tendency of prices is maintained. A fair business has been done in menthol at higher prices; for some considerable time the market for this drug had been very quiet, and the tendency of the price was in a downward direction, but now that the article has begun to attract interest again we may see still higher prices. A fair business has been done in gum benzoin. Cream of tartar is still very scarce, and the price continues to rise. The value of salicylic acid and sodium salicylate is well maintained. There is a fairly good demand for senna, and prices have advanced slightly. Persian opium is again dearer, and in view of the scarcity it is improbable that the highest figure has yet been reached.

### TO OUR READERS.

**Contributions are specially invited from any of our readers to these columns. They should deal with topical subjects and news. They must be authenticated for the information of the Editor only. The minimum payment if published is 5s. There is no hard-and-fast rule as to space, but notes of about twenty lines in length are preferred.**

